# Immediate Postoperative Low Platelet Counts After Living Donor Liver Transplantation Predict Early Allograft Dysfunction

**DOI:** 10.1097/MD.0000000000001373

**Published:** 2015-08-28

**Authors:** Lei Li, Haiqing Wang, Jian Yang, Li Jiang, Jiayin Yang, Wentao Wang, Lvnan Yan, Tianfu Wen, Bo Li, Mingqing Xu

**Affiliations:** From the Department of Liver Surgery, Liver Transplantation Center, West China Hospital of Sichuan University, Chengdu, Sichuan Province, China (LL, JY, LJ, JY, WW, LY, TW, BL, MX); and Department of Hepato-Biliary-Pancreatic Surgery, Sichuan Cancer Hospital, Chengdu, Sichuan Province, China (HW).

## Abstract

To investigate whether the platelets can improve liver function by mediating liver regeneration. Using a retrospective cohort with 234 consecutive adult-to-adult living donor liver transplantation recipients, we have discussed the relationship between immediate postoperative platelet count and outcome. Patients have been stratified into Low Platelet Group (106 patients) with platelet ≤68 × 10^9^/L and High Platelet Group (128 patients) with platelet >68 × 10^9^/L.

Low Platelet Group has a higher rate of preoperative thrombocytopenia (90.6% vs. 32.8%, *P* < 0.001), higher model for end-stage liver disease score (15 vs. 11, *P* < 0.001), cirrhosis (86.8% vs. 76.6%, *P* = 0.046), hepatorenal syndrome (18.2% vs. 4.0%, *P* = 0.005) and fulminant hepatic failure (26.4% vs. 7.8%, *P* < 0.001). The packed red blood cells transfusion (7.5 vs. 5, *P* = 0.023) and plasma transfusion (1275 mL vs. 800 mL, *P* = 0.001) are more in patients with low platelet count. Low Platelet Group has a higher early allograft dysfunction (EAD) (22.6% vs. 7.0%, *P* = 0.001) and severe complications (22.6% vs. 10.9%, *P* = 0.016). The 90-day mortality between the 2 groups is similar. The multivariate analysis has found that postoperative platelet ≤68 × 10^9^/L is an independent risk factor for EAD.

Platelet maybe influences the functional status of the liver by promoting graft regeneration after liver transplantation.

## INTRODUCTION

Liver transplantation (LT) has been accepted as the only effective and standard therapeutic modality for patients with end-stage liver disease. Living donor liver transplantation (LDLT) expands the donor pool and thus alleviates the problem of organ shortage.^1^ With many technical principles of refinement and standardization, the regeneration rate of graft with undersized graft is a major concern for most surgeons after LDLT.^2^ Partial liver grafts need a rapid regeneration to meet the functional demands for recipients, or liver failure would happen and the short- and long-term outcomes would be affected. Up to now, comparatively little information is available on the factors affecting human liver regeneration, except for graft size, ischemia time, and portal venous flow or pressure.^2^ Recent animals and clinical studies have demonstrated that platelets do play a role not only in blood coagulation,^3^ tissue repair,^4^ and ischemia/reperfusion injury,^5^ but also in liver regeneration.^6–13^ Platelet transfusion after LDLT has also been confirmed to be related to liver regeneration.^2^ Moreover, immediate postoperative low platelet count has recently been proved to be associated with delayed liver function recovery after partial liver resection for colorectal liver metastases, which has indicated that platelets play a critical role in liver regeneration after liver resection.^14^ However, the role of immediate postoperative low platelet count in LT recipients with living donor has not been investigated. We have embarked on the present study to determine the relationship between immediate postoperative platelet count and outcome after adult-to-adult LDLT (A-A LDLT).

## MATERIALS AND METHODS

### Study Population

Between May 2002 and January 2014, a total of 320 consecutive LDLT were performed at West China Hospital. We excluded recipients aged less than 18 years (67 recipients) and recipients who had platelet transfusion after LT (19 recipients) in our present study. This resulted in a total of 234 patients included in our study. The cohort was stratified into 2 groups according to the immediate postoperative platelet count. Clinical and demographic data of donors and recipients were collected from the records of the Chinese Liver Transplant Registry (CLTR: http://cltr.cotr.cn). The protocol was approved by the West China Hospital Ethical Committee and written informed consents were obtained from all the recipients before their operation. Preoperative and postoperative platelet counts were prospectively recorded daily from admission until postoperative day 7, weekly from postoperative week 1 until week 4, monthly from month 1 to month 3. Follow-up information was collected at least 3 months after transplantation. The donor selection, surgical procedure (for donor and recipients), and posttransplant management were as those previously described.^15^

### Outcome Parameters

The primary outcome measure was early allograft dysfunction (EAD), defined as the presence of one or more of the following postoperative laboratory: bilirubin ≥10 mg/dL on day 7, international normalized ratio ≥1.6 on day 7, and alanine or aspartate aminotransferases >2000 IU/L within the first 7 days.^16^ The Clavien–Dindo complication classification^17^ system was used for postoperative complication grading and grade III–IV complications were defined as severe complications. Mortality was defined as any death occurring from the time of surgery up to 90 days after transplantation.^18^ Primary graft nonfunction was defined as death or retransplantation within the first postoperative week after the exclusion of technical, immunological, and infectious causes.^1,18,19^ The immediate postoperative platelet count was defined as the platelet count obtained immediately after surgery, usually upon the arrival at the intensive care unit (ICU) after LT surgery.

### Statistical Analysis

All statistical analyses were performed using SPSS Version 17 statistical software, and statistical significance was set at *P* < 0.05. Continuous variables were reported as mean (SD) or median (range), and were compared using the Student *t* test for continuous variables with parametric distribution, Mann–Whitney *U* test or Kruskal–Wallis *H* test for those with nonparametric distribution. Categorical variables were reported as numbers and percentages, and compared using Pearson χ^2^ analysis or Fisher exact test. The predictive ability of immediate postoperative low platelet count for EAD was assessed by the receiver operating characteristic (ROC) curve and corresponding area under the ROC (AUROC) curve. The optimal cutoff value was set as the value maximizing the sum of sensitivity and specificity, namely Youden index.^18^ To identify risk factors for EAD and severe complications, only significant factors associated with EAD and severe complications in the univariate analysis were entered into the forward stepwise logistic regression analysis.

## RESULTS

### Patient Characteristics and in the Low Platelet Group and High Platelet Group

**FIGURE 1:**
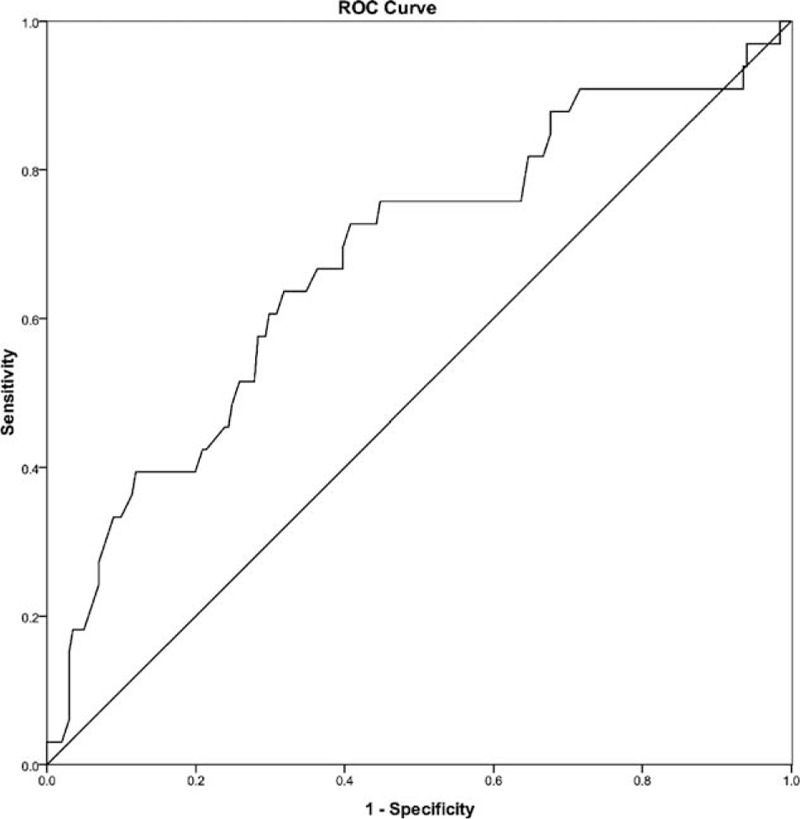
ROC curve for immediate postoperative platelet count in relation to postoperative early allograft dysfunction (area under the curve = 0.678, *P* < 0.001). The platelet count of 68 × 10^9^/L was the most accurate cutoff value with the highest Youden index (Youden index = 0.319, sensitivity = 0.727, specificity = 0.592) for predicting postoperative early allograft dysfunction. ROC = the receiver operating characteristic.

**FIGURE 2:**
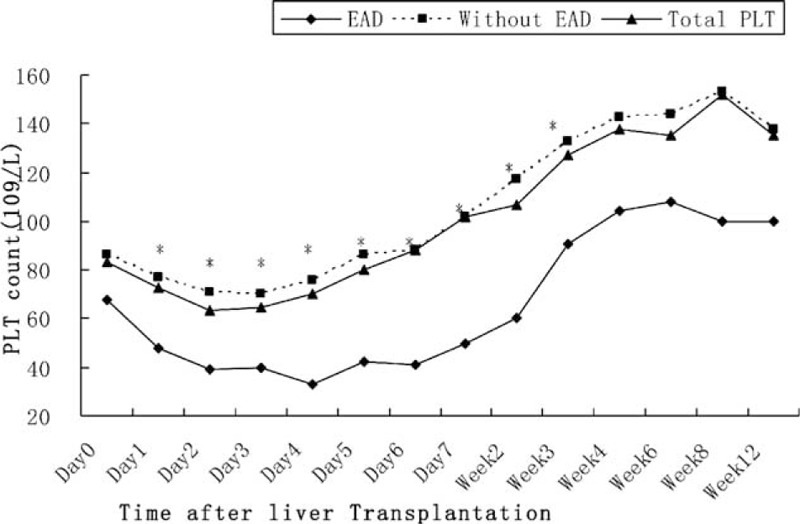
Platelet count changes after liver transplantation. EAD = early allograft dysfunction.

A total of 234 recipients undergoing A-A LDLT were included in our study. Pretransplant diagnosis included 106 hepatocellular carcinoma, 39 fulminant liver failure, 45 hepatitis B cirrhosis, 8 hepatocholangiocarcinoma, 4 Budd–Chiari syndrome, 3 retransplantation, 11 biliary cirrhosis, 6 alcoholic cirrhosis, 5 cirrhosis with hepatitis C virus, 2 hepatic hydatidosis, and 5 others. Three recipients underwent dual donors LT. Of the remaining 231 patients, 213 (91.0%) recipients accepted right lobe of liver without middle hepatic vein, 12 (5.1%) recipients accepted right lobe of liver with middle hepatic vein and 6 (2.6%) recipients accepted left lobe of liver. The median pretransplant platelet count was 83 × 10^9^/L and the lowest median platelet count after LT was 64 × 10^9^/L on postoperative day 2. Based on AUROC curve, immediate postoperative platelet count showed a good prediction ability (Figure 1) (AUROC = 0.678, *P* < 0.001) for EAD. The optimal cutoff value for prediction EAD was 68 × 10^9^/L with the maximizing Youden index of 0.319 (sensitivity = 0.727, specificity = 0.592). With this cut-off value, patients were stratified into the Low Platelet Group (platelet count ≤68 × 10^9^/L) with 106 recipients and High Platelet Group with 128 recipients (platelet count >68 × 10^9^/L). After an obvious decrease during the first 2 days after LT, a persistent increasing in platelet count was observed until 12 weeks after operation, not only in the whole cohort, but also in the patients with EAD and without EAD (Figure 2). Patients with EAD had a significant lower platelet counts than patients without EAD from postoperative day 1 to weeks 3.

The patients characteristics of the 2 groups are shown in Table 1. Patients in the Low Platelet Group had a higher rate of preoperative thrombocytopenia (90.6% vs. 32.8%, *P* < 0.001), a higher model for end-stage liver disease (MELD) score (15 vs. 11, *P* < 0.001), more packed red blood cells (PRBCs) transfusion (7.5 U vs. 5 U, *P* = 0.023), and more plasma transfusion (1275 mL vs. 800 mL, *P* = 0.001). In addition, Low Platelet Group had a significantly higher incidence of hepatitis B surface antigen (HBsAg) (84% vs. 69.5%, *P* = 0.01), cirrhosis (86.8% vs. 76.6%, *P* = 0.046), hepatorenal syndrome (18.2% vs. 4.0%, *P* = 0.005), and fulminant hepatic failure (26.4% vs. 7.8%, *P* < 0.001). There were no significant differences between the 2 groups regarding the other analyzed parameters (Table 1).

**TABLE 1:**
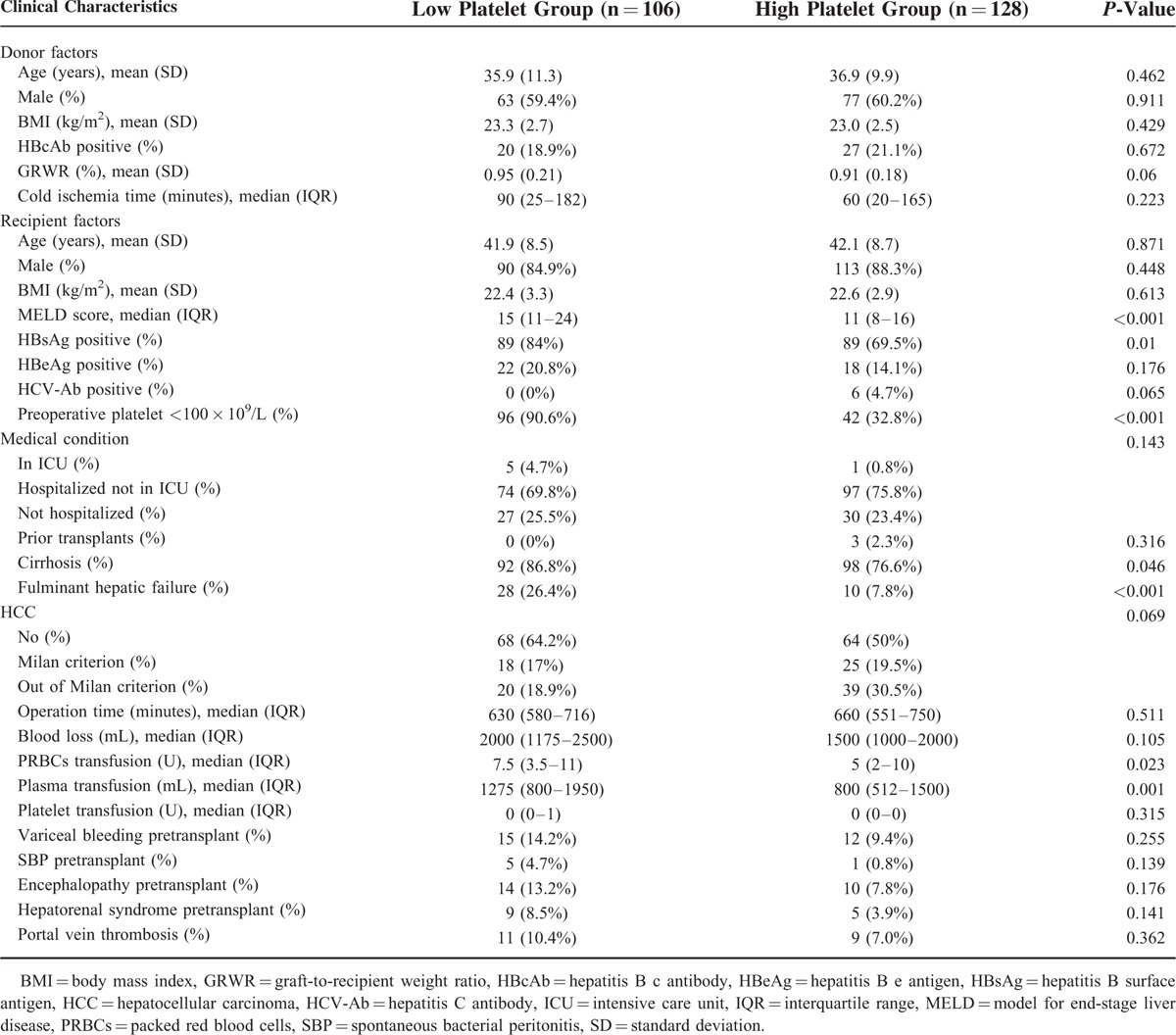
Characteristic Between Patients With Low or High Platelet Count After Liver Transplantation

### Postoperative Outcomes in the Low Platelet Group and High Platelet Group

In our cohort, there were 38 patients with severe complications and 31 recipients died during the first 3 months after LT (Table 2). The morbidity of severe complications was 16.2% and the 90-day mortality was 13.2%. Thirty-three (14.1%) patients suffered from EAD and only 2 recipients were diagnosed as primary liver nonfunction. Compared to the High Platelet Group, the Low Platelet Group had more severe complications (22.6% vs. 10.9%, *P* = 0.016) and EAD. EAD in the Low Platelet Group was 22.6%, which was at least 3 times higher than that in the High Platelet Group with a rate of 7.0% (*P* < 0.001). Although Low Platelet Group seemed have a higher 90-day mortality (16% vs. 10.9%), the differences did not reach statistical significance (*P* = 0.252). No significant difference was observed in primary liver nonfunction and ICU stay time between the 2 groups. In addition, we also analyzed the relationship between preoperative platelet count and postoperative outcomes and found that preoperative thrombocytopenia was associated with EAD (77.8% vs. 55.7%, *P* = 0.013), not with severe complications and the 90-day mortality. The mean follow-up was 24 months and the overall patient (*P* = 0.85) and graft survival rates (*P* = 0.91) were similar in both age groups.

**TABLE 2:**
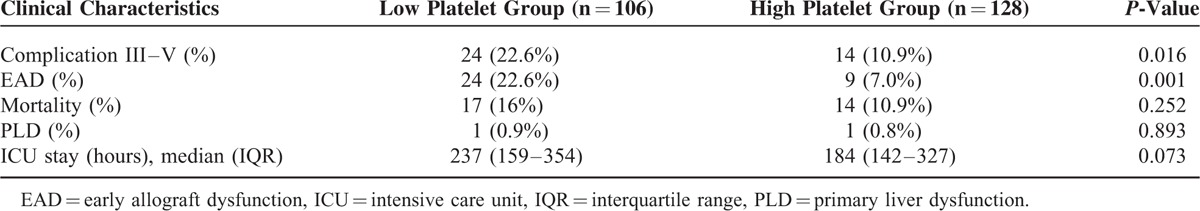
Postoperative Outcome Between Patients With Low or High Platelet Count After Liver Transplantation

### Risk factors for EAD and Severe Complications

In order to identify the risk factors for postoperative EAD, a univariate analysis of patients with and without postoperative EAD was carried out. Univariate analysis (Table 3) showed that 6 variables, including postoperative platelet count ≤68 × 10^9^/L, were significantly associated with the occurrence of postoperative EAD. The 6 variables were postoperative platelet count ≤68 × 10^9^/L, preoperative platelet, hepatorenal syndrome pretransplant, plasma transfusion, fulminant hepatic failure, and MELD score. These significantly different variables were included in a multivariate logistic regression model to identify whether postoperative platelet count ≤68 × 10^9^/L is an independent risk factor for EAD. The logistic regression analysis (Table 4) indicated that fulminant hepatic failure, postoperative platelet count ≤68 × 10^9^/L and hepatorenal syndrome pretransplant were independent risk factors for EAD. Preoperative platelet is not an independent risk factors, but low platelet count remained a strong and independent risk factor for EAD, with the OR of 2.88 (95% CI, 1.22–6.82; *P* = 0.016).

**TABLE 3:**
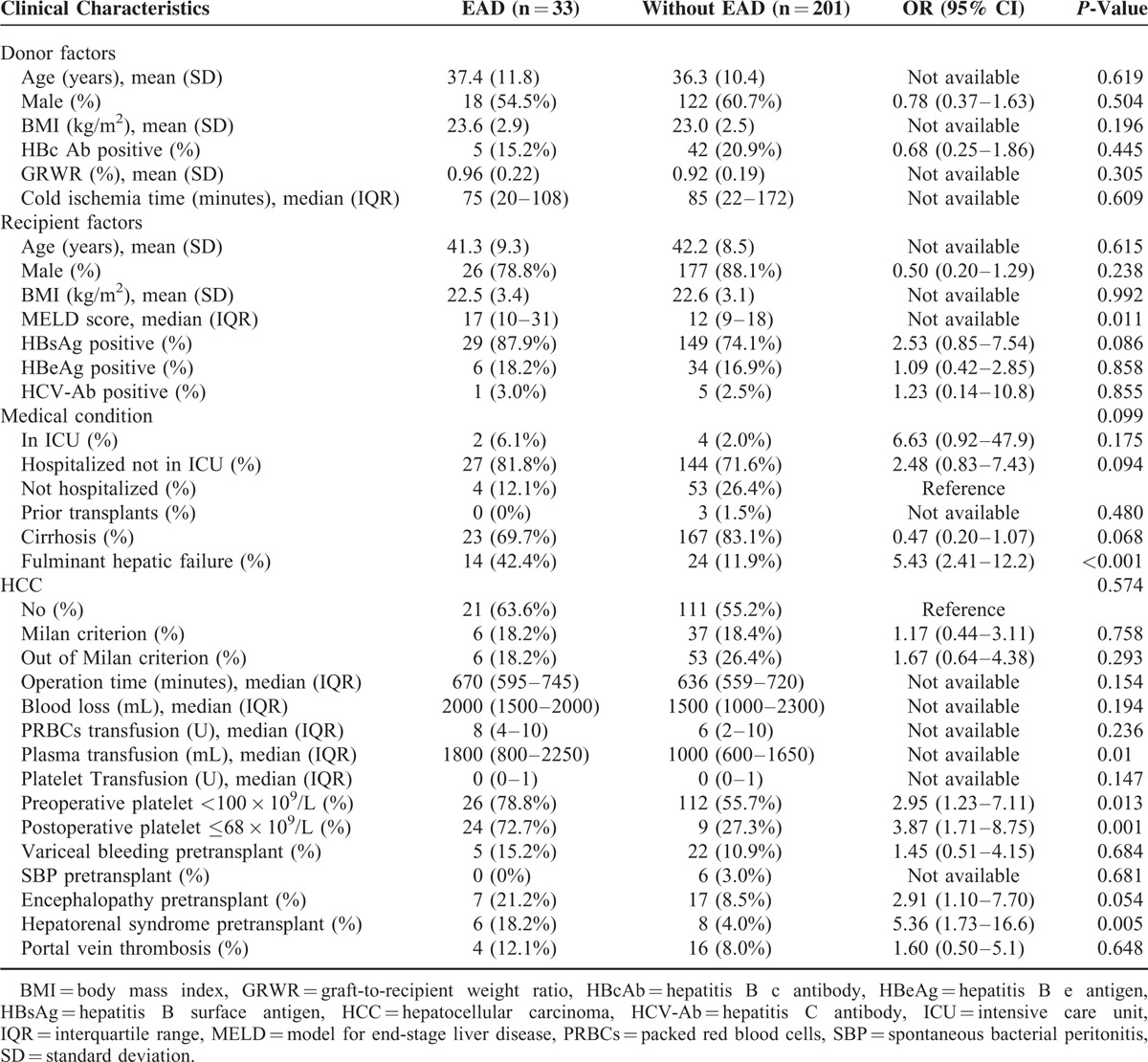
Characteristic Between Patients With or Without EAD After Liver Transplantation

**TABLE 4:**
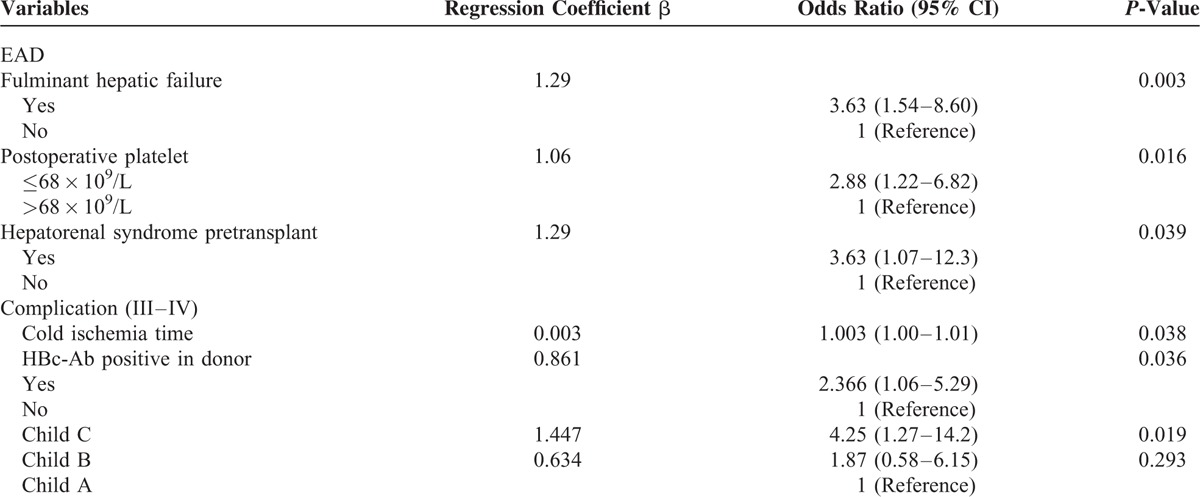
Multivariate Analysis of Independent Risk Factor for EAD and Postoperative Complication

In addition, we also identified whether low platelet count was an independent risk factor for postoperative severe complications. Five variables, including cold ischemia time, donor hepatitis B c antibody positive, fulminant hepatic failure, Child-Pugh classification, and postoperative platelet count ≤68 × 10^9^/L were identified as the risk factors for postoperative severe complications by univariate analysis. The logistic regression analysis (Table 4) indicated that Child-Pugh classification C, cold ischemia time, and hepatitis B c antibody positive in donor were independent risk factors. Postoperative platelet count ≤68 × 10^9^/L was not an independent risk factor for postoperative severe complications.

## DISCUSSION

EAD is a major factor influencing the morbidity and mortality after LT, especially for LDLT recipients in whom the small-for-size grafts must regenerate to adjust to the recipient's condition for preventing from liver failure. Many preoperative and intra-operative parameters^20^ affecting EAD have been evaluated, however, the effect of postoperative low platelet count on postoperative EAD and morbidity is not well known. We have carried out a cohort study to assess whether immediate postoperative low platelet count affect postoperative outcomes of LDLT. In our study, recipients with immediate postoperative low platelet count are associated with an increased frequency of EAD and severe complications. This has demonstrated that low platelet count is an independent predictor of postoperative EAD.

LT is the most effective treatment for end-stage liver disease and hepatocellular carcinoma patients, the majority of whom are always accompanied with various degrees of cirrhosis. Preoperative thrombocytopenia, related to the degree of liver fibrosis, is a frequent comorbidity mainly deriving from portal hypertension-related splenic sequestration in the cirrhotic liver. There is not only the well-known feature of thrombocytopenia, but also decreased platelet function in cirrhosis patients.^3^ The negative effect of preoperative thrombocytopenia on postoperative outcomes has been confirmed by several studies. According to these studies, preoperative thrombocytopenia is not only associated with postoperative ascite,^10^ increased mortality and morbidity,^11–13^ but also is a risk factor for survival in both hepatocellular carcinoma^21^ and LT patients.^22^

However, a reduction in the platelet count occurs more frequently immediately after LT. Many studies have found that the platelet count has reached a nadir at days 2 to 5 posttransplant but returned to preoperative levels by weeks 1 to 2.^18,23–25^ Although the mechanisms of fall in postoperative platelet are not completely understood,^24^ many factors have been identified to be associated with thrombocytopenia, including low preoperative platelet,^23^ sequestration of the platelets in the reperfused liver graft, platelet consumption, impaired production of thrombopoietin,^24^ hemodilution and thrombin generation.^25^ Up to now, although several reports^24–31^ have been published on discussing the postoperative low platelet count, they have mainly focused on the changes and risk factors for postoperative thrombocytopenia. Rare studies have focused on evaluating the role of postoperative thrombocytopenia. Chang et al^26^ has found that persistent thrombocytopenia would portend a higher rate of fungal infections in liver transplant recipients. Another interesting study is from Lesurtel et al^18^ and he has proposed a criterion called “60-5 criterion” to predict the occurrence of severe complications after LT. The author has conducted a study including 257 consecutive deceased cardiac LT recipients and has found that platelet count <60 × 10^9^/L on postoperative day 5 is associated with severe complications, EAD, and graft survival. These finding have indicated that platelet does play a critical role on liver regeneration after LT. However, the timing limitation of the criterion (postoperative day 5) for predicting complications has made the application of the criterion less available. Even if a patient is suspected for complications according to this criterion, the measure cannot be in time taken for prevention or the complication has already happened. That is because the majority of the severe complications have occurred shortly after LT.

Therefore, we have conducted a cohort including 234 A-A LDLT recipients to evaluate whether immediate postoperative platelet count can predict the postoperative outcomes. Now that low platelet can result in negative effects mainly by inadequate liver regeneration and postischemic liver repair mechanisms,^18^ EAD, not severe complications, is the best choice as the primary outcome. In addition, only A-A LDLT have been included in our cohort because these recipients with part grafts require more liver regeneration than deceased whole graft to meet the function of metabolic, synthetic, and detoxification requirements in liver.

In our study, recipients with low platelet had a higher MELD score, higher incidence of HBsAg, cirrhosis, hepatorenal syndrome, and fulminant hepatic failure. These parameters were mainly associated with the degree of liver fibrosis from hepatitis B/C infection, and they also reflected the preoperative platelet count. Additionally, Low Platelet Group had more PRBCs transfusion and more plasma transfusion, and these 2 factors reflected the consumption of platelet during operation. Not surprisingly, the Low Platelet Group did have more EAD and severe complications. This seemed to suggest a cascade of events resulting in a domino effect of patients with poorer conditions in Low Platelet Group and having more complicated surgery and an expected complicated postoperative course.^32^ But the multivariate analysis found that postoperative platelet ≤68 × 10^9^/L and another 2 factors (fulminant hepatic failure and hepatorenal syndrome) were independent risk factors for EAD. Low platelet count was proved to be a strong and independent risk factor for EAD, with the OR of 2.88. But, postoperative platelet ≤68 × 10^9^/L was not an independent risk factor for postoperative severe complications. The results suggested that immediate postoperative low platelet mainly affected the recovery of the liver function by liver regeneration, and had no direct effect on total postoperative severe complications. In addition, the role of platelet on liver regeneration has been confirmed in liver resection. Alkozai^14^ reported a series of 216 patients with liver resection and hence demonstrated that a low immediate postoperative platelet count was an independent predictor of delayed postoperative liver function recovery and was associated with an increased risk of postoperative mortality.

Accumulating evidences from experimental and clinical studies have indicated that platelets do not only play a role in hemostasis and thrombogenesis, but can also improve liver function by mediating liver regeneration.^6^ Recent animal experiments have suggested that platelets, or rather platelet-derived serotonin, contribute to cell cycle progression and metabolic pathways to prevent acute liver failure.^4,6,8–9^ Other studies have also proved that thrombopoietin^8^ or platelets infused via the portal vein^7^ can stimulate regeneration after hepatectomy in rats. This phenomenon derived from animal experiments has also been confirmed in clinical practice. A retrospective study^2^ has showed that transfused platelets are significantly associated with graft regeneration in liver donors.

In general, our results are in line with the “60-5 criterion,” except for predicting survival and some details. Though the primary outcomes, cut-off values and donor types are different between the 2 studies, they have all confirmed that the postoperative low platelet after LT are associated with worse short-term outcome. Our findings are of significance for physician to take positive measures to increase platelet count to prevent EAD after LT. For recipients with postoperative low platelet, preventive measure such as platelet transfusion, administration of thrombopoietin and serotonin, and withdrawal of drugs of potential myelosuppression, should be taken in time to promote liver regeneration. However, further research is needed before this treatment can be considered for use in the clinic.

Several limitations have existed in our study. First, the degree of persistent errhysis from the liver donor may obviously affect the postoperative platelet count. Second, our study is a retrospective cohort with limited simple, which would reduce the strength of argument. We look forward to prospective clinical investigations to evaluate the role of platelet.

In conclusion, we have conducted a retrospective study on A-A LDLT and found that immediate postoperative platelet count ≤68 × 10^9^/L was an independent risk factor for postoperative EAD. Platelet maybe influences the functional status of the liver by promoting graft regeneration after LT. However, further prospective studies are required to assess the role of the immediate postoperative low platelet count in LT patients.
